# Koetjapic acid chloro­form hemisolvate

**DOI:** 10.1107/S1600536810016430

**Published:** 2010-05-08

**Authors:** Z. D. Nassar, A. F. A. Aisha, A. M. S. Abdul Majid, Chin Sing Yeap, Hoong-Kun Fun

**Affiliations:** a Department of Pharmacology, School of Pharmaceutical Sciences, Universiti Sains Malaysia, 11800 USM, Penang, Malaysia; b X-ray Crystallography Unit, School of Physics, Universiti Sains Malaysia, 11800 USM, Penang, Malaysia

## Abstract

The asymmetric unit of the title compound, C_30_H_46_O_4_·0.5CHCl_3_, consists of one koetjapic acid [systematic name: (3*R*,4a*R*,4b*S*,7*S*,8*S*,10b*S*,12a*S*)-7-(2-carboxy­ethyl)-3,4b,7,10b,12a-penta­methyl-8-(prop-1-en-2-yl)-1,2,3,4,4a,4b,5,6,7,8,9,10,10b,11,12,12a-hexa­deca­hydro­chrysene-3-carboxylic acid] mol­ecule and one half-mol­ecule of chloro­form solvent, which is disordered about a twofold rotation axis. The symmetry-independent component is further disordered over two sites, with occupancies of 0.30 and 0.20. The koetjapic acid contains a fused four-ring system, *A*/*B*/*C*/*D*. The *A*/*B*, *B*/*C* and *C*/*D* junctions adopt *E*/*trans*/*cis* configurations, respectively. The conformation of ring *A* is inter­mediate between envelope and half-chair and ring *B* adopts an envelope conformation whereas rings *C* and *D* adopt chair conformations. A weak intra­molecular C—H⋯O hydrogen bond is observed. The koetjapic acid mol­ecules are linked into dimers by two pairs of inter­molecular O—H⋯O hydrogen bonds. The dimers are stacked along the *c* axis.

## Related literature

For the biological properties of *Sandoricum koetjape* and koetjapic acid, see: Aisha *et al.* (2009 ▶); Kaneda *et al.* (1992 ▶); Sun *et al.* (1999 ▶); Ismail *et al.* (2003 ▶); Rasadah *et al.* (2004 ▶). For ring puckering parameters, see: Cremer & Pople (1975 ▶). For the stability of the temperature controller used in the data collection, see: Cosier & Glazer (1986 ▶).
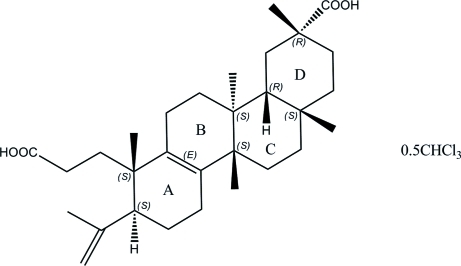


## Experimental

### 

#### Crystal data

C_30_H_46_O_4_·0.5CHCl_3_
*M* *_r_* = 530.35
Orthorhombic, 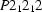

*a* = 12.8950 (3) Å
*b* = 33.7309 (8) Å
*c* = 6.5307 (1) Å
*V* = 2840.59 (10) Å^3^
*Z* = 4
Cu *K*α radiation
μ = 1.88 mm^−1^
*T* = 100 K
0.34 × 0.28 × 0.11 mm

#### Data collection

Bruker APEXII DUO CCD area-detector diffractometer
Absorption correction: multi-scan (*SADABS*; Bruker, 2009 ▶) *T*_min_ = 0.567, *T*_max_ = 0.827
26063 measured reflections
4654 independent reflections
4467 reflections with *I* > 2σ(*I*)
*R*_int_ = 0.031

#### Refinement

*R*[*F* ^2^ > 2σ(*F* ^2^)] = 0.060
*wR*(*F* ^2^) = 0.169
*S* = 1.09
4654 reflections
357 parameters
40 restraints
H-atom parameters constrained
Δρ_max_ = 0.53 e Å^−3^
Δρ_min_ = −0.68 e Å^−3^
Absolute structure: Flack (1983 ▶), 1876 Friedel pairs
Flack parameter: 0.08 (5)

### 

Data collection: *APEX2* (Bruker, 2009 ▶); cell refinement: *SAINT* (Bruker, 2009 ▶); data reduction: *SAINT*; program(s) used to solve structure: *SHELXTL* (Sheldrick, 2008 ▶); program(s) used to refine structure: *SHELXTL*; molecular graphics: *SHELXTL*; software used to prepare material for publication: *SHELXTL* and *PLATON* (Spek, 2009 ▶).

## Supplementary Material

Crystal structure: contains datablocks global, I. DOI: 10.1107/S1600536810016430/ci5066sup1.cif

Structure factors: contains datablocks I. DOI: 10.1107/S1600536810016430/ci5066Isup2.hkl

Additional supplementary materials:  crystallographic information; 3D view; checkCIF report

## Figures and Tables

**Table 1:** Hydrogen-bond geometry (Å, °)

*D*—H⋯*A*	*D*—H	H⋯*A*	*D*⋯*A*	*D*—H⋯*A*
O1—H1⋯O4^i^	0.82	1.81	2.621 (6)	168
O3—H3⋯O2^i^	0.82	1.85	2.670 (4)	176
C22—H22*B*⋯O1	0.96	2.56	3.380 (5)	144

Symmetry code: (i) 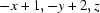.

**Fig. 1.**
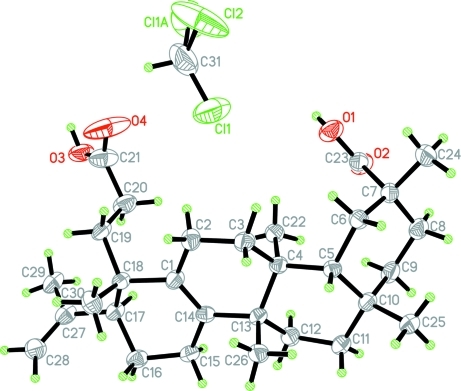
The molecular structure of koetjapic acid, showing 50% probability displacement ellipsoids and the atom-numbering scheme. Only one disorder component of the chloroform solvent molecule is shown. Atom Cl1A is generated by the symmetry operation (1-x, 2-y, z).

**Fig. 2.**
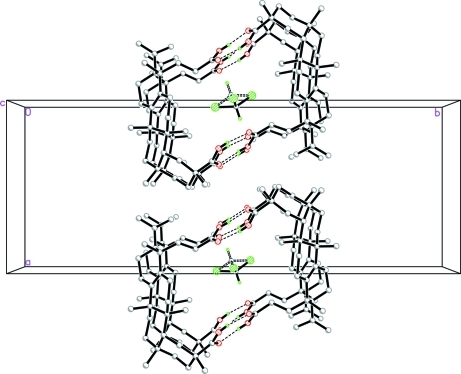
Part of the crystal packing of the title compound, viewed along the c axis, showing dimers stacked along the c axis. Hydrogen bonds are shown as dashed lines. For clarity, Only two disorder components of the chloroform solvent molecule are shown.

## supplementary crystallographic information

### Comment

*n*-Hexane extract of *Sandoricum koetjape* was reported previously to have cytotoxic and apoptotic properties on HCT-116 colon cancer cell line (Aisha *et al.*, 2009). The koetjapic acid can be isolated from the stem bark of *S. koetjape* using column chromatography and was studied for cytotoxic activity (Kaneda *et al.*, 1992). This compound was found to possess DNA polymerase β inhibition (Sun *et al.*, 1999), ichthyotoxic (Ismail *et al.*, 2003), and anti-inflammatory properties (Rasadah *et al.*, 2004). The structure of koetjapic acid was established previously by several research groups from spectral evidence (Kaneda *et al.*, 1992; Rasadah *et al.*, 2004; Sun *et al.*, 1999). Herein we describe a new simple method to purify koetjapic acid from *Sandoricum koetjape*. This method utilizes two steps of crystallization process but without the use of column chromatography.

The asymmetric unit of title compound, consists of one koetjapic acid molecule and half a molecule of disordered chloroform solvent (Fig. 1). The koetjapic acid contains fused four-ring system, A/B/C/D (Scheme 1). The A/B, B/C and C/D junctions adopt E/trans/cis configuration, respectively. The conformation of the ring A is intermediate between envelope and half-chair [Q = 0.516 (4) Å, θ = 131.6 (3)°, φ = 45.7 (5)°]. The ring B adopts an envelope conformation [Q = 0.509 (3) Å, θ = 52.9 (3)°, φ = 177.6 (5)°] whereas ring C and D adopt chair conformations [Q = 0.561 (3) Å, θ = 155.6 (3)°, φ = 117.8 (8)°; Q = 0.491 (4) Å, θ = 168.1 (5)°, φ = 77 (2)°] (Cremer & Pople, 1975). A weak intramolecular C22—H22B···O1 hydrogen bond is observed in the molecular structure.

The koetjapic acid molecules are linked into dimers by two pairs of intermolecular O1—H1···O4 and O3—H3···O2 hydrogen bonds (Table 1). The dimers are stacked along the *c* axis (Fig. 2).

The chloroform molecule is disordered over four sites in a void with twofold symmetry. In one set of symmetry-related sites, atom Cl2 lies on the twofold rotation axis and the three Cl sites [Cl1, Cl2, Cl1A; occupancy 0.60] are shared by two disorder components. In the other set of symmetry-related site, atoms Cl3 and Cl3A [occupancy 0.40] are shared by two disorder components with atoms Cl4 or Cl4A [occupancy 0.20] in each component. The chloroform solvent molecule lies in the cavity produced by the dimer.

### Experimental

The title compound was isolated from the stem bark of *Sandoricum koetjape*, which was collected from the main campus of Universiti Sains Malaysia on middle of October 2009. 200 g of the dried powder was extracted with 1000 ml of n-hexane at 313 K for 24 hours with intermittent shaking. The extract was filtered and concentrated to dryness under reduced pressure at 313 K to give 10 g of solid material. 10 g of the extract was dissolved in 50 ml, 1:1, methanol-acetone and was kept at 253 K. After 24 hours, a white precipitate was formed. The solid was filtered and washed thrice with ice-chilled chloroform. The compound was crystallized from chloroform to give colourless prism-shaped crystals (400 mg). Single crystals suitable for X-ray analysis were obtained by slow evaporation of a chloroform solution at room temperature.

### Refinement

All H atoms were positioned geometrically and refined using a riding model, with C–H = 0.93–0.98 Å, *U*_iso_(H) = 1.2 or 1.5 *U*_eq_(C) and O–H = 0.82 Å, *U*_iso_(H) = 1.5 *U*_eq_(O). A rotating group model was applied for the methyl groups. The chloroform molecule is disordered across a twofold rotation axis. The symmetry independent component is further disordered over two sites with occupancies of 0.30 and 0.20. All C—Cl distances were restrained to be equal and U^ij^ components of Cl atoms were restrained to an approximate isotropic behaviour. The distance between atoms Cl3 and Cl4 at (1-x, 2-y, z) were restrained to 2.60 (1) Å. The highest residual electron density peak is located at 0.53 Å from Cl3 and the deepest hole is located at 0.68 Å from Cl2. 1876 Friedel pairs were used to determine the absolute configuration using the anomalous scattering of the CuKα radiation.

### Crystal data

**Table:**

C_30_H_46_O_4_·0.5CHCl_3_	*F*(000) = 1148
*M**_r_* = 530.35	*D*_x_ = 1.240 Mg m^−^^3^
Orthorhombic, *P*2_1_2_1_2	Cu *K*α radiation, λ = 1.54178 Å
Hall symbol: P 2 2ab	Cell parameters from 9931 reflections
*a* = 12.8950 (3) Å	θ = 2.6–65.7°
*b* = 33.7309 (8) Å	µ = 1.88 mm^−^^1^
*c* = 6.5307 (1) Å	*T* = 100 K
*V* = 2840.59 (10) Å^3^	Block, colourless
*Z* = 4	0.34 × 0.28 × 0.11 mm

### Data collection

**Table:**

Bruker APEXII DUO CCD area-detector diffractometer	4654 independent reflections
Radiation source: fine-focus sealed tube	4467 reflections with *I* > 2σ(*I*)
graphite	*R*_int_ = 0.031
φ and ω scans	θ_max_ = 66.1°, θ_min_ = 2.6°
Absorption correction: multi-scan (*SADABS*; Bruker, 2009)	*h* = −15→15
*T*_min_ = 0.567, *T*_max_ = 0.827	*k* = −38→39
26063 measured reflections	*l* = −5→7

### Refinement

**Table:**

Refinement on *F*^2^	Hydrogen site location: inferred from neighbouring sites
Least-squares matrix: full	H-atom parameters constrained
*R*[*F*^2^ > 2σ(*F*^2^)] = 0.060	*w* = 1/[σ^2^(*F*_o_^2^) + (0.0958*P*)^2^ + 1.9305*P*] where *P* = (*F*_o_^2^ + 2*F*_c_^2^)/3
*wR*(*F*^2^) = 0.169	(Δ/σ)_max_ = 0.001
*S* = 1.09	Δρ_max_ = 0.53 e Å^−^^3^
4654 reflections	Δρ_min_ = −0.68 e Å^−^^3^
357 parameters	Extinction correction: *SHELXTL* (Sheldrick, 2008), Fc^*^=kFc[1+0.001xFc^2^λ^3^/sin(2θ)]^-1/4^
40 restraints	Extinction coefficient: 0.0082 (8)
Primary atom site location: structure-invariant direct methods	Absolute structure: Flack (1983), 1876 Friedel pairs
Secondary atom site location: difference Fourier map	Flack parameter: 0.08 (5)

### Special details

**Table:**

Experimental. The crystal was placed in the cold stream of an Oxford Cryosystems Cobra open-flow nitrogen cryostat (Cosier & Glazer, 1986) operating at 100.0 (1) K.
Geometry. All esds (except the esd in the dihedral angle between two l.s. planes) are estimated using the full covariance matrix. The cell esds are taken into account individually in the estimation of esds in distances, angles and torsion angles; correlations between esds in cell parameters are only used when they are defined by crystal symmetry. An approximate (isotropic) treatment of cell esds is used for estimating esds involving l.s. planes.
Refinement. Refinement of *F*^2^ against ALL reflections. The weighted *R*-factor wR and goodness of fit *S* are based on *F*^2^, conventional *R*-factors *R* are based on *F*, with *F* set to zero for negative *F*^2^. The threshold expression of *F*^2^ > σ(*F*^2^) is used only for calculating *R*-factors(gt) etc. and is not relevant to the choice of reflections for refinement. *R*-factors based on *F*^2^ are statistically about twice as large as those based on *F*, and *R*- factors based on ALL data will be even larger.

### Fractional atomic coordinates and isotropic or equivalent isotropic displacement parameters (Å^2^)

**Table:**

	*x*	*y*	*z*	*U*_iso_*/*U*_eq_	Occ. (<1)
O1	0.2093 (2)	0.96882 (8)	0.3593 (5)	0.0439 (8)	
H1	0.2417	0.9868	0.4147	0.066*	
O2	0.1261 (2)	0.96828 (8)	0.6601 (5)	0.0436 (8)	
O3	0.7713 (3)	0.96911 (9)	0.8069 (6)	0.0514 (9)	
H3	0.8000	0.9887	0.7590	0.077*	
O4	0.6996 (5)	0.96705 (14)	0.4969 (7)	0.106 (2)	
C1	0.5175 (3)	0.84482 (11)	0.6074 (6)	0.0304 (8)	
C2	0.4878 (3)	0.86704 (12)	0.4167 (6)	0.0329 (9)	
H2A	0.5314	0.8577	0.3056	0.039*	
H2B	0.5037	0.8949	0.4378	0.039*	
C3	0.3751 (3)	0.86394 (12)	0.3479 (6)	0.0328 (9)	
H3A	0.3678	0.8412	0.2578	0.039*	
H3B	0.3572	0.8875	0.2700	0.039*	
C4	0.2988 (3)	0.85964 (10)	0.5262 (6)	0.0262 (8)	
C5	0.1868 (3)	0.85387 (10)	0.4384 (6)	0.0264 (8)	
H5A	0.1946	0.8317	0.3427	0.032*	
C6	0.1433 (3)	0.88781 (10)	0.3023 (6)	0.0292 (8)	
H6A	0.2020	0.9006	0.2369	0.035*	
H6B	0.1027	0.8755	0.1944	0.035*	
C7	0.0758 (3)	0.92092 (11)	0.3973 (7)	0.0339 (9)	
C8	0.0003 (3)	0.90414 (12)	0.5567 (7)	0.0391 (10)	
H8A	−0.0273	0.9259	0.6374	0.047*	
H8B	−0.0574	0.8918	0.4859	0.047*	
C9	0.0491 (3)	0.87389 (12)	0.7005 (7)	0.0354 (9)	
H9A	−0.0044	0.8636	0.7905	0.042*	
H9B	0.1000	0.8873	0.7851	0.042*	
C10	0.1020 (3)	0.83885 (11)	0.5927 (6)	0.0300 (9)	
C11	0.1466 (3)	0.81017 (12)	0.7559 (6)	0.0356 (9)	
H11A	0.1008	0.8107	0.8737	0.043*	
H11B	0.1444	0.7835	0.7001	0.043*	
C12	0.2567 (3)	0.81809 (12)	0.8308 (6)	0.0325 (9)	
H12A	0.2788	0.7966	0.9190	0.039*	
H12B	0.2578	0.8424	0.9103	0.039*	
C13	0.3322 (3)	0.82188 (10)	0.6488 (5)	0.0252 (8)	
C14	0.4463 (3)	0.82387 (10)	0.7164 (5)	0.0254 (8)	
C15	0.4753 (3)	0.79887 (11)	0.8945 (6)	0.0303 (8)	
H15A	0.4413	0.7734	0.8803	0.036*	
H15B	0.4484	0.8113	1.0176	0.036*	
C16	0.5911 (3)	0.79172 (12)	0.9237 (6)	0.0346 (9)	
H16A	0.6142	0.7710	0.8312	0.042*	
H16B	0.6043	0.7830	1.0628	0.042*	
C17	0.6514 (3)	0.82981 (11)	0.8812 (6)	0.0304 (8)	
H17A	0.6197	0.8505	0.9658	0.036*	
C18	0.6348 (3)	0.84244 (11)	0.6529 (6)	0.0270 (8)	
C19	0.6891 (3)	0.88239 (12)	0.6111 (6)	0.0335 (9)	
H19A	0.7628	0.8790	0.6352	0.040*	
H19B	0.6803	0.8888	0.4673	0.040*	
C20	0.6517 (4)	0.91792 (12)	0.7379 (7)	0.0440 (11)	
H20A	0.5779	0.9218	0.7178	0.053*	
H20B	0.6640	0.9130	0.8823	0.053*	
C21	0.7097 (5)	0.95397 (14)	0.6711 (8)	0.0539 (13)	
C22	0.3104 (3)	0.89708 (11)	0.6604 (6)	0.0325 (9)	
H22A	0.2509	0.8996	0.7478	0.049*	
H22B	0.3157	0.9200	0.5742	0.049*	
H22C	0.3718	0.8948	0.7427	0.049*	
C23	0.1407 (3)	0.95455 (11)	0.4877 (7)	0.0338 (9)	
C24	0.0136 (4)	0.94111 (12)	0.2222 (8)	0.0453 (11)	
H24A	0.0608	0.9509	0.1205	0.068*	
H24B	−0.0260	0.9627	0.2770	0.068*	
H24C	−0.0324	0.9221	0.1610	0.068*	
C25	0.0193 (3)	0.81565 (11)	0.4729 (7)	0.0378 (9)	
H25A	−0.0317	0.8055	0.5663	0.057*	
H25B	0.0515	0.7940	0.4017	0.057*	
H25C	−0.0136	0.8329	0.3758	0.057*	
C26	0.3264 (3)	0.78339 (11)	0.5175 (6)	0.0326 (9)	
H26A	0.3786	0.7842	0.4128	0.049*	
H26B	0.2592	0.7815	0.4551	0.049*	
H26C	0.3378	0.7607	0.6036	0.049*	
C27	0.7649 (3)	0.82688 (13)	0.9473 (6)	0.0357 (9)	
C28	0.8203 (3)	0.79269 (13)	0.9245 (7)	0.0440 (11)	
H28A	0.8880	0.7912	0.9730	0.066*	
H28B	0.7904	0.7709	0.8604	0.066*	
C29	0.8118 (3)	0.86138 (13)	1.0484 (6)	0.0407 (10)	
H29A	0.8764	0.8538	1.1099	0.061*	
H29B	0.7657	0.8711	1.1523	0.061*	
H29C	0.8240	0.8818	0.9491	0.061*	
C30	0.6836 (3)	0.81256 (12)	0.5030 (6)	0.0361 (9)	
H30A	0.6690	0.8205	0.3649	0.054*	
H30B	0.6549	0.7867	0.5271	0.054*	
H30C	0.7573	0.8118	0.5236	0.054*	
C31	0.5335 (9)	0.9947 (3)	0.0839 (17)	0.087 (5)	0.50
H31A	0.6041	0.9856	0.0890	0.104*	0.299 (3)
H31B	0.5871	0.9780	0.1384	0.104*	0.201 (3)
Cl1	0.4660 (3)	0.95921 (9)	0.1615 (9)	0.1061 (18)	0.597 (7)
Cl2	0.5000	1.0000	−0.2013 (6)	0.127 (3)	0.597 (7)
Cl3	0.4269 (6)	0.9672 (3)	−0.0147 (14)	0.153 (4)	0.403 (7)
Cl4	0.4584 (9)	1.0233 (3)	0.2864 (15)	0.107 (4)	0.201 (3)

### Atomic displacement parameters (Å^2^)

**Table:**

	*U*^11^	*U*^22^	*U*^33^	*U*^12^	*U*^13^	*U*^23^
O1	0.0454 (17)	0.0302 (14)	0.0561 (18)	−0.0102 (13)	0.0042 (15)	−0.0052 (14)
O2	0.0436 (17)	0.0284 (14)	0.059 (2)	−0.0053 (12)	0.0067 (15)	−0.0103 (14)
O3	0.0545 (19)	0.0332 (15)	0.067 (2)	−0.0172 (14)	−0.0117 (17)	0.0117 (15)
O4	0.166 (5)	0.075 (3)	0.076 (3)	−0.080 (3)	−0.044 (3)	0.034 (2)
C1	0.0276 (19)	0.033 (2)	0.0302 (19)	−0.0056 (16)	−0.0014 (16)	0.0033 (15)
C2	0.030 (2)	0.044 (2)	0.0252 (19)	0.0015 (17)	0.0039 (16)	0.0049 (16)
C3	0.033 (2)	0.036 (2)	0.030 (2)	−0.0023 (16)	−0.0003 (16)	0.0074 (16)
C4	0.0269 (18)	0.0229 (17)	0.0287 (18)	−0.0009 (14)	0.0005 (15)	−0.0021 (14)
C5	0.0267 (18)	0.0226 (17)	0.0298 (19)	−0.0008 (14)	−0.0016 (15)	−0.0012 (14)
C6	0.0297 (19)	0.0253 (18)	0.033 (2)	−0.0020 (15)	−0.0062 (16)	−0.0020 (15)
C7	0.0256 (19)	0.0235 (18)	0.053 (2)	0.0005 (15)	−0.0041 (19)	−0.0037 (17)
C8	0.030 (2)	0.0284 (19)	0.059 (3)	−0.0028 (17)	0.0018 (19)	−0.0086 (18)
C9	0.027 (2)	0.036 (2)	0.043 (2)	−0.0044 (16)	0.0065 (17)	−0.0050 (18)
C10	0.0264 (18)	0.0276 (19)	0.036 (2)	−0.0038 (15)	0.0027 (16)	−0.0006 (16)
C11	0.030 (2)	0.036 (2)	0.041 (2)	−0.0039 (17)	0.0063 (18)	0.0087 (17)
C12	0.031 (2)	0.036 (2)	0.031 (2)	0.0012 (16)	0.0034 (16)	0.0055 (17)
C13	0.0256 (18)	0.0250 (17)	0.0250 (17)	−0.0030 (14)	0.0028 (14)	0.0002 (14)
C14	0.031 (2)	0.0205 (17)	0.0246 (17)	−0.0027 (14)	−0.0020 (14)	−0.0041 (14)
C15	0.034 (2)	0.0255 (18)	0.031 (2)	−0.0047 (16)	0.0044 (17)	0.0018 (15)
C16	0.038 (2)	0.033 (2)	0.033 (2)	−0.0072 (18)	−0.0078 (17)	0.0074 (16)
C17	0.0301 (19)	0.0321 (19)	0.0288 (18)	−0.0076 (16)	−0.0003 (16)	0.0018 (15)
C18	0.0269 (19)	0.0263 (18)	0.0277 (18)	−0.0041 (15)	0.0005 (15)	0.0024 (14)
C19	0.030 (2)	0.036 (2)	0.035 (2)	−0.0099 (16)	0.0028 (17)	0.0069 (16)
C20	0.048 (3)	0.032 (2)	0.052 (3)	−0.0112 (19)	0.004 (2)	−0.0015 (19)
C21	0.075 (4)	0.033 (2)	0.054 (3)	−0.014 (2)	−0.013 (3)	0.005 (2)
C22	0.032 (2)	0.0271 (19)	0.038 (2)	−0.0031 (16)	−0.0067 (17)	−0.0032 (16)
C23	0.030 (2)	0.0221 (18)	0.050 (2)	0.0003 (15)	−0.0005 (19)	−0.0031 (18)
C24	0.037 (2)	0.031 (2)	0.067 (3)	0.0004 (18)	−0.012 (2)	0.003 (2)
C25	0.031 (2)	0.028 (2)	0.054 (2)	−0.0047 (17)	−0.0031 (19)	−0.0002 (18)
C26	0.032 (2)	0.0250 (18)	0.041 (2)	−0.0009 (15)	−0.0048 (17)	−0.0022 (16)
C27	0.037 (2)	0.043 (2)	0.0267 (19)	−0.0099 (18)	−0.0052 (16)	0.0083 (17)
C28	0.036 (2)	0.042 (2)	0.054 (3)	−0.0047 (19)	−0.013 (2)	0.009 (2)
C29	0.037 (2)	0.050 (3)	0.035 (2)	−0.0166 (19)	−0.0029 (18)	0.0055 (18)
C30	0.036 (2)	0.040 (2)	0.032 (2)	0.0063 (17)	−0.0022 (17)	−0.0023 (17)
C31	0.066 (8)	0.051 (7)	0.143 (13)	0.020 (6)	−0.016 (8)	−0.002 (8)
Cl1	0.103 (3)	0.0606 (17)	0.154 (4)	−0.0130 (16)	0.036 (3)	0.032 (2)
Cl2	0.245 (7)	0.078 (3)	0.059 (2)	0.095 (4)	0.000	0.000
Cl3	0.154 (6)	0.141 (6)	0.162 (7)	−0.086 (5)	0.055 (5)	−0.070 (6)
Cl4	0.151 (8)	0.077 (5)	0.094 (6)	−0.008 (6)	0.001 (6)	−0.038 (5)

### Geometric parameters (Å, °)

**Table:**

O1—C23	1.310 (5)	C17—C27	1.529 (6)
O1—H1	0.82	C17—C18	1.565 (5)
O2—C23	1.232 (5)	C17—H17A	0.98
O3—C21	1.296 (6)	C18—C30	1.539 (5)
O3—H3	0.82	C18—C19	1.543 (5)
O4—C21	1.227 (6)	C19—C20	1.534 (6)
C1—C14	1.360 (5)	C19—H19A	0.97
C1—C2	1.504 (5)	C19—H19B	0.97
C1—C18	1.544 (5)	C20—C21	1.492 (6)
C2—C3	1.525 (6)	C20—H20A	0.97
C2—H2A	0.97	C20—H20B	0.97
C2—H2B	0.97	C22—H22A	0.96
C3—C4	1.531 (5)	C22—H22B	0.96
C3—H3A	0.97	C22—H22C	0.96
C3—H3B	0.97	C24—H24A	0.96
C4—C22	1.545 (5)	C24—H24B	0.96
C4—C13	1.565 (5)	C24—H24C	0.96
C4—C5	1.565 (5)	C25—H25A	0.96
C5—C6	1.554 (5)	C25—H25B	0.96
C5—C10	1.571 (5)	C25—H25C	0.96
C5—H5A	0.98	C26—H26A	0.96
C6—C7	1.546 (5)	C26—H26B	0.96
C6—H6A	0.97	C26—H26C	0.96
C6—H6B	0.97	C27—C28	1.365 (6)
C7—C23	1.528 (5)	C27—C29	1.468 (6)
C7—C8	1.534 (6)	C28—H28A	0.93
C7—C24	1.554 (6)	C28—H28B	0.93
C8—C9	1.523 (6)	C29—H29A	0.96
C8—H8A	0.97	C29—H29B	0.96
C8—H8B	0.97	C29—H29C	0.96
C9—C10	1.536 (5)	C30—H30A	0.96
C9—H9A	0.97	C30—H30B	0.96
C9—H9B	0.97	C30—H30C	0.96
C10—C25	1.538 (6)	C31—C31^i^	0.94 (2)
C10—C11	1.550 (5)	C31—Cl4^i^	1.460 (13)
C11—C12	1.525 (6)	C31—Cl3^i^	1.526 (10)
C11—H11A	0.97	C31—Cl1	1.564 (9)
C11—H11B	0.97	C31—Cl1^i^	1.635 (9)
C12—C13	1.543 (5)	C31—Cl3	1.779 (11)
C12—H12A	0.97	C31—Cl4	1.903 (12)
C12—H12B	0.97	C31—Cl2	1.920 (12)
C13—C14	1.538 (5)	C31—H31A	0.96
C13—C26	1.558 (5)	C31—H31B	0.96
C14—C15	1.484 (5)	Cl1—C31^i^	1.635 (9)
C15—C16	1.525 (6)	Cl1—H31B	1.6921
C15—H15A	0.97	Cl2—C31^i^	1.920 (12)
C15—H15B	0.97	Cl3—C31^i^	1.526 (10)
C16—C17	1.527 (5)	Cl4—C31^i^	1.460 (13)
C16—H16A	0.97	Cl4—Cl4^i^	1.91 (2)
C16—H16B	0.97		
			
C23—O1—H1	109.5	H16A—C16—H16B	108.2
C21—O3—H3	109.5	C16—C17—C27	112.4 (3)
C14—C1—C2	121.4 (3)	C16—C17—C18	109.4 (3)
C14—C1—C18	122.3 (3)	C27—C17—C18	114.7 (3)
C2—C1—C18	115.8 (3)	C16—C17—H17A	106.6
C1—C2—C3	116.9 (3)	C27—C17—H17A	106.6
C1—C2—H2A	108.1	C18—C17—H17A	106.6
C3—C2—H2A	108.1	C30—C18—C19	105.9 (3)
C1—C2—H2B	108.1	C30—C18—C1	108.2 (3)
C3—C2—H2B	108.1	C19—C18—C1	111.4 (3)
H2A—C2—H2B	107.3	C30—C18—C17	111.8 (3)
C2—C3—C4	113.3 (3)	C19—C18—C17	110.1 (3)
C2—C3—H3A	108.9	C1—C18—C17	109.3 (3)
C4—C3—H3A	108.9	C20—C19—C18	116.4 (3)
C2—C3—H3B	108.9	C20—C19—H19A	108.2
C4—C3—H3B	108.9	C18—C19—H19A	108.2
H3A—C3—H3B	107.7	C20—C19—H19B	108.2
C3—C4—C22	107.0 (3)	C18—C19—H19B	108.2
C3—C4—C13	106.8 (3)	H19A—C19—H19B	107.3
C22—C4—C13	110.4 (3)	C21—C20—C19	108.8 (4)
C3—C4—C5	109.0 (3)	C21—C20—H20A	109.9
C22—C4—C5	113.5 (3)	C19—C20—H20A	109.9
C13—C4—C5	109.9 (3)	C21—C20—H20B	109.9
C6—C5—C4	116.8 (3)	C19—C20—H20B	109.9
C6—C5—C10	110.7 (3)	H20A—C20—H20B	108.3
C4—C5—C10	116.6 (3)	O4—C21—O3	123.9 (5)
C6—C5—H5A	103.5	O4—C21—C20	120.7 (5)
C4—C5—H5A	103.5	O3—C21—C20	115.4 (4)
C10—C5—H5A	103.5	C4—C22—H22A	109.5
C7—C6—C5	120.4 (3)	C4—C22—H22B	109.5
C7—C6—H6A	107.2	H22A—C22—H22B	109.5
C5—C6—H6A	107.2	C4—C22—H22C	109.5
C7—C6—H6B	107.2	H22A—C22—H22C	109.5
C5—C6—H6B	107.2	H22B—C22—H22C	109.5
H6A—C6—H6B	106.9	O2—C23—O1	123.4 (4)
C23—C7—C8	111.1 (4)	O2—C23—C7	123.2 (4)
C23—C7—C6	112.5 (3)	O1—C23—C7	113.3 (4)
C8—C7—C6	111.3 (3)	C7—C24—H24A	109.5
C23—C7—C24	104.0 (3)	C7—C24—H24B	109.5
C8—C7—C24	109.5 (3)	H24A—C24—H24B	109.5
C6—C7—C24	108.2 (4)	C7—C24—H24C	109.5
C9—C8—C7	113.8 (3)	H24A—C24—H24C	109.5
C9—C8—H8A	108.8	H24B—C24—H24C	109.5
C7—C8—H8A	108.8	C10—C25—H25A	109.5
C9—C8—H8B	108.8	C10—C25—H25B	109.5
C7—C8—H8B	108.8	H25A—C25—H25B	109.5
H8A—C8—H8B	107.7	C10—C25—H25C	109.5
C8—C9—C10	114.6 (4)	H25A—C25—H25C	109.5
C8—C9—H9A	108.6	H25B—C25—H25C	109.5
C10—C9—H9A	108.6	C13—C26—H26A	109.5
C8—C9—H9B	108.6	C13—C26—H26B	109.5
C10—C9—H9B	108.6	H26A—C26—H26B	109.5
H9A—C9—H9B	107.6	C13—C26—H26C	109.5
C9—C10—C25	108.5 (3)	H26A—C26—H26C	109.5
C9—C10—C11	109.3 (3)	H26B—C26—H26C	109.5
C25—C10—C11	106.8 (3)	C28—C27—C29	120.2 (4)
C9—C10—C5	110.8 (3)	C28—C27—C17	121.7 (4)
C25—C10—C5	108.7 (3)	C29—C27—C17	118.0 (4)
C11—C10—C5	112.6 (3)	C27—C28—H28A	120.0
C12—C11—C10	117.1 (3)	C27—C28—H28B	120.0
C12—C11—H11A	108.0	H28A—C28—H28B	120.0
C10—C11—H11A	108.0	C27—C29—H29A	109.5
C12—C11—H11B	108.0	C27—C29—H29B	109.5
C10—C11—H11B	108.0	H29A—C29—H29B	109.5
H11A—C11—H11B	107.3	C27—C29—H29C	109.5
C11—C12—C13	110.8 (3)	H29A—C29—H29C	109.5
C11—C12—H12A	109.5	H29B—C29—H29C	109.5
C13—C12—H12A	109.5	C18—C30—H30A	109.5
C11—C12—H12B	109.5	C18—C30—H30B	109.5
C13—C12—H12B	109.5	H30A—C30—H30B	109.5
H12A—C12—H12B	108.1	C18—C30—H30C	109.5
C14—C13—C12	112.8 (3)	H30A—C30—H30C	109.5
C14—C13—C26	103.9 (3)	H30B—C30—H30C	109.5
C12—C13—C26	109.0 (3)	Cl4^i^—C31—Cl3^i^	135.2 (9)
C14—C13—C4	112.0 (3)	Cl1—C31—Cl1^i^	129.0 (8)
C12—C13—C4	106.7 (3)	Cl4^i^—C31—Cl3	99.6 (7)
C26—C13—C4	112.5 (3)	Cl3^i^—C31—Cl3	123.0 (9)
C1—C14—C15	122.4 (3)	Cl4^i^—C31—Cl4	67.6 (10)
C1—C14—C13	121.2 (3)	Cl3^i^—C31—Cl4	92.0 (6)
C15—C14—C13	116.2 (3)	Cl3—C31—Cl4	97.1 (6)
C14—C15—C16	115.7 (3)	Cl1—C31—Cl2	105.1 (6)
C14—C15—H15A	108.3	Cl1^i^—C31—Cl2	102.3 (6)
C16—C15—H15A	108.3	Cl1—C31—H31A	105.8
C14—C15—H15B	108.3	Cl1^i^—C31—H31A	106.8
C16—C15—H15B	108.3	Cl2—C31—H31A	106.1
H15A—C15—H15B	107.4	Cl4^i^—C31—H31B	50.8
C15—C16—C17	110.0 (3)	Cl3^i^—C31—H31B	114.2
C15—C16—H16A	109.7	Cl3—C31—H31B	112.6
C17—C16—H16A	109.7	Cl4—C31—H31B	114.0
C15—C16—H16B	109.7	Cl2—C31—H31B	125.2
C17—C16—H16B	109.7		
			
C14—C1—C2—C3	−1.8 (6)	C15—C16—C17—C18	−62.5 (4)
C18—C1—C2—C3	169.8 (3)	C14—C1—C18—C30	97.3 (4)
C1—C2—C3—C4	32.3 (5)	C2—C1—C18—C30	−74.3 (4)
C2—C3—C4—C22	60.6 (4)	C14—C1—C18—C19	−146.7 (4)
C2—C3—C4—C13	−57.6 (4)	C2—C1—C18—C19	41.7 (5)
C2—C3—C4—C5	−176.3 (3)	C14—C1—C18—C17	−24.7 (5)
C3—C4—C5—C6	−60.2 (4)	C2—C1—C18—C17	163.7 (3)
C22—C4—C5—C6	58.9 (4)	C16—C17—C18—C30	−66.4 (4)
C13—C4—C5—C6	−176.9 (3)	C27—C17—C18—C30	61.0 (4)
C3—C4—C5—C10	165.8 (3)	C16—C17—C18—C19	176.2 (3)
C22—C4—C5—C10	−75.1 (4)	C27—C17—C18—C19	−56.5 (4)
C13—C4—C5—C10	49.1 (4)	C16—C17—C18—C1	53.4 (4)
C4—C5—C6—C7	−94.0 (4)	C27—C17—C18—C1	−179.2 (3)
C10—C5—C6—C7	42.5 (4)	C30—C18—C19—C20	178.2 (4)
C5—C6—C7—C23	85.3 (4)	C1—C18—C19—C20	60.8 (5)
C5—C6—C7—C8	−40.2 (5)	C17—C18—C19—C20	−60.7 (5)
C5—C6—C7—C24	−160.5 (3)	C18—C19—C20—C21	−177.8 (4)
C23—C7—C8—C9	−82.4 (4)	C19—C20—C21—O4	64.4 (7)
C6—C7—C8—C9	43.8 (5)	C19—C20—C21—O3	−113.9 (5)
C24—C7—C8—C9	163.3 (3)	C8—C7—C23—O2	−6.2 (5)
C7—C8—C9—C10	−55.0 (5)	C6—C7—C23—O2	−131.8 (4)
C8—C9—C10—C25	−63.0 (4)	C24—C7—C23—O2	111.5 (5)
C8—C9—C10—C11	−179.1 (3)	C8—C7—C23—O1	177.5 (3)
C8—C9—C10—C5	56.3 (4)	C6—C7—C23—O1	52.0 (5)
C6—C5—C10—C9	−47.5 (4)	C24—C7—C23—O1	−64.8 (4)
C4—C5—C10—C9	89.1 (4)	C16—C17—C27—C28	37.4 (5)
C6—C5—C10—C25	71.6 (4)	C18—C17—C27—C28	−88.4 (5)
C4—C5—C10—C25	−151.8 (3)	C16—C17—C27—C29	−139.0 (4)
C6—C5—C10—C11	−170.3 (3)	C18—C17—C27—C29	95.2 (4)
C4—C5—C10—C11	−33.6 (4)	Cl4^i^—C31—Cl1—C31^i^	116.2 (8)
C9—C10—C11—C12	−88.7 (4)	Cl3^i^—C31—Cl1—C31^i^	−23 (4)
C25—C10—C11—C12	154.1 (4)	Cl1^i^—C31—Cl1—C31^i^	48.8 (10)
C5—C10—C11—C12	34.9 (5)	Cl3—C31—Cl1—C31^i^	−58.6 (5)
C10—C11—C12—C13	−52.2 (5)	Cl4—C31—Cl1—C31^i^	48.8 (6)
C11—C12—C13—C14	−171.7 (3)	Cl2—C31—Cl1—C31^i^	−71.3 (5)
C11—C12—C13—C26	−56.9 (4)	Cl4^i^—C31—Cl2—C31^i^	90 (3)
C11—C12—C13—C4	64.9 (4)	Cl3^i^—C31—Cl2—C31^i^	−95.7 (11)
C3—C4—C13—C14	55.0 (4)	Cl1—C31—Cl2—C31^i^	72.3 (11)
C22—C4—C13—C14	−60.9 (4)	Cl1^i^—C31—Cl2—C31^i^	−64.2 (9)
C5—C4—C13—C14	173.1 (3)	Cl3—C31—Cl2—C31^i^	62.1 (10)
C3—C4—C13—C12	178.9 (3)	Cl4—C31—Cl2—C31^i^	−18.6 (7)
C22—C4—C13—C12	63.0 (4)	Cl4^i^—C31—Cl3—C31^i^	99.5 (8)
C5—C4—C13—C12	−63.0 (4)	Cl3^i^—C31—Cl3—C31^i^	−65.9 (11)
C3—C4—C13—C26	−61.7 (4)	Cl1—C31—Cl3—C31^i^	103.9 (6)
C22—C4—C13—C26	−177.6 (3)	Cl1^i^—C31—Cl3—C31^i^	−6.6 (11)
C5—C4—C13—C26	56.4 (4)	Cl4—C31—Cl3—C31^i^	31.2 (7)
C2—C1—C14—C15	174.5 (3)	Cl2—C31—Cl3—C31^i^	−90.1 (6)
C18—C1—C14—C15	3.5 (6)	Cl4^i^—C31—Cl4—C31^i^	−134.0 (15)
C2—C1—C14—C13	0.2 (6)	Cl3^i^—C31—Cl4—C31^i^	87.1 (11)
C18—C1—C14—C13	−170.9 (3)	Cl1—C31—Cl4—C31^i^	−79.0 (12)
C12—C13—C14—C1	−148.5 (4)	Cl1^i^—C31—Cl4—C31^i^	101.0 (12)
C26—C13—C14—C1	93.6 (4)	Cl3—C31—Cl4—C31^i^	−36.5 (11)
C4—C13—C14—C1	−28.1 (5)	Cl2—C31—Cl4—C31^i^	24.5 (8)
C12—C13—C14—C15	36.8 (4)	C31^i^—C31—Cl4—Cl4^i^	134.0 (15)
C26—C13—C14—C15	−81.0 (4)	Cl3^i^—C31—Cl4—Cl4^i^	−138.9 (10)
C4—C13—C14—C15	157.2 (3)	Cl1—C31—Cl4—Cl4^i^	55.0 (7)
C1—C14—C15—C16	−11.2 (5)	Cl1^i^—C31—Cl4—Cl4^i^	−125.0 (10)
C13—C14—C15—C16	163.4 (3)	Cl3—C31—Cl4—Cl4^i^	97.5 (8)
C14—C15—C16—C17	40.8 (5)	Cl2—C31—Cl4—Cl4^i^	158.5 (9)
C15—C16—C17—C27	168.9 (3)		

Symmetry codes: (i) −*x*+1, −*y*+2, *z*.

### Hydrogen-bond geometry (Å, °)

**Table:**

*D*—H···*A*	*D*—H	H···*A*	*D*···*A*	*D*—H···*A*
O1—H1···O4^i^	0.82	1.81	2.621 (6)	168
O3—H3···O2^i^	0.82	1.85	2.670 (4)	176
C22—H22B···O1	0.96	2.56	3.380 (5)	144

Symmetry codes: (i) −*x*+1, −*y*+2, *z*.
